# The Molecular Epidemiology and Evolutionary Dynamics of Influenza B Virus in Two Italian Regions during 2010–2015: The Experience of Sicily and Liguria

**DOI:** 10.3390/ijms17040549

**Published:** 2016-04-13

**Authors:** Fabio Tramuto, Andrea Orsi, Carmelo Massimo Maida, Claudio Costantino, Cecilia Trucchi, Cristiano Alicino, Francesco Vitale, Filippo Ansaldi

**Affiliations:** 1 Department of Health Promotion Sciences and Mother-Child Care “G. D’Alessandro”—Hygiene Section, University of Palermo, Palermo 90127, Italy; carmelo.maida@unipa.it (C.M.M.); claudio.costantino01@unipa.it (C.C.); francesco.vitale@unipa.it (F.V.); 2 Clinical Epidemiology Unit, University Hospital “Paolo Giaccone”, Palermo 90127, Italy; 3 Department of Health Sciences, University of Genoa, Genoa 16132, Italy; andrea.orsi@unige.it (A.O.); cecilia.trucchi@edu.unige.it (C.T.); cristiano.alicino@unige.it (C.A.); filippo.ansaldi@unige.it (F.A.); 4 Hygiene Unit, IRCCS University Hospital “San Martino”—IST National Institute for Cancer Research, Genoa 16132, Italy

**Keywords:** influenza B, lineages, viral evolution, surveillance, molecular epidemiology, influenza-like illness, Sicily, Liguria, Italy

## Abstract

Molecular epidemiology of influenza B virus remained poorly studied in Italy, despite representing a major contributor to seasonal epidemics. This study aimed to reconstruct the phylogenetic relationships and genetic diversity of the hemagglutinin gene sequences of 197 influenza B strains circulating in both Southern (Sicily) and Northern (Liguria) Italy between 2010 and 2015. Upper respiratory tract specimens of patients displaying symptoms of influenza-like illness were screened by real-time RT-PCR assay for the presence of influenza B virus. PCR-positive influenza B samples were further analyzed by sequencing. Neighbor-joining phylogenetic trees were constructed and the amino-acid alignments were analyzed. Phylogenetic analysis showed clusters in B/Victoria clade 1A/1B (*n* = 29, 14.7%), and B/Yamagata clades 2 (*n* = 112, 56.8%) and 3 (*n* = 56, 28.4%). Both influenza B lineages were found to co-circulate during the study period, although a lineage swap from B/Victoria to B/Yamagata occurred in Italy between January 2011 and January 2013. The most represented amino-acid substitutions were N116K in the 120-loop (83.9% of B/Yamagata clade 3 strains) and I146V in the 150-loop (89.6% of B/Victoria clade 1 strains). D197N in 190-helix was found in almost all viruses collected. Our findings provide further evidence to support the adoption of quadrivalent influenza vaccines in our country.

## 1. Introduction

Influenza is a respiratory infectious disease that affects humans and is essentially sustained by influenza viruses type A and B that cause serious outbreaks worldwide during cold season [1,2,3]. The two influenza types usually co-circulate during seasonal epidemics and relative proportions significantly differ each year (Figure S1).

Influenza A virus infects humans, swine, birds, and horses, whereas influenza B virus seems to affect humans only, even though its presence has been confirmed in oropharyngeal swabs obtained from seals [4,5] and domestic pigs [6].

Around twenty influenza A subtypes are actually known, a few of which are responsible for seasonal epidemics in humans. Conversely, influenza B is not formally classified into subtypes leading to a lower genetic diversity as compared to type A. Viral adaptation and evolution of influenza B have been limited to various mechanisms of deletion, insertion or intra-host genetic reassortment of hemagglutinin (HA)/neuraminidase segments in co-infected subjects [7].

The first isolated strain of influenza B was B/Lee/40 [8]. Since 1983, influenza B strains evolved into two distinct major lineages that vary considerably, both antigenically and genetically: B/Victoria/2/87-like (Victoria-lineage) and B/Yamagata/16/88-like (Yamagata-lineage) [9].

The Victoria-lineage predominated during the 1980s, while the Yamagata-lineage prevailed in the most part of the world during the 1990s [10]. In 2001, Victoria-lineage strains re-emerged in Europe and United States and, because of demographics and rapid movements of human populations between countries, the two lineages have co-circulated since then [11].

Despite the burden influenza B has demonstrated globally [2,12], scientific studies concerning the epidemiology of influenza viruses have mainly focused on influenza A and, in Italy, the knowledge of circulating influenza B strains is still very limited [13,14].

Molecular surveillance of influenza viruses covers almost the entire globe. It is essential to monitor the impact of infection on community health and to follow the genetic evolutionary pattern and antigenic variability of viral strains in order to adapt the yearly production of influenza vaccines.

In this regard, the co-circulation of two distinct lineages of influenza B viruses and the low level of cross-protection against strains of the opposite B-lineage may limit the efficacy of the current trivalent vaccine formulations [15,16], adding evidence to support the widespread adoption of quadrivalent vaccines [17,18].

This study aimed to phylogenetically investigate the heterogeneity of influenza B viruses detected over the period 2010–2015 in two different Italian administrative regions located in the South (Sicily) and in the North (Liguria) of the country.

## 2. Results

A total of 197 HA gene sequences collected in Italy between 2010 and 2015 from 288 patients with influenza-like illness symptoms testing positive for influenza B virus were phylogenetically analyzed.

**Figure 1:**
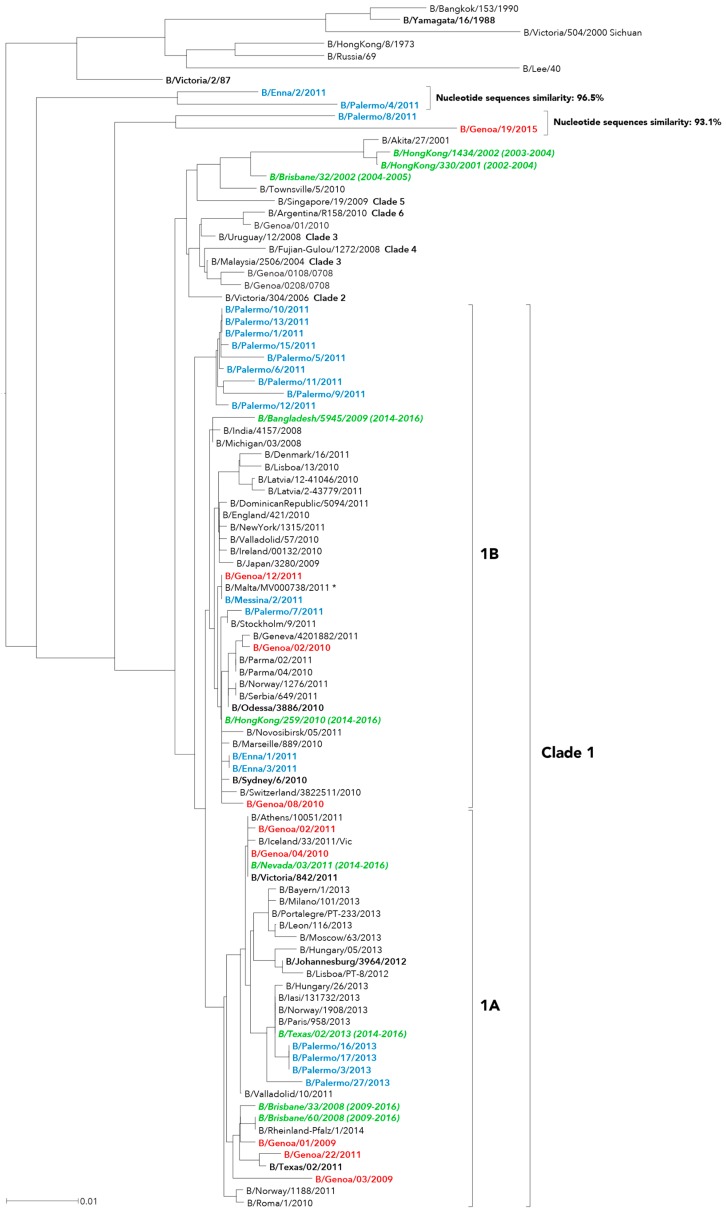
Phylogenetic analysis of the hemagglutinin (HA) nucleotide sequences from influenza B Victoria-lineage viruses isolated in Italy from 2010 to 2015. The phylogeny tree was generated by the neighbor-joining method with 1000 bootstrap replicates. Sicilian strains (blue color), Ligurian strains (red color), WHO recommended candidate vaccine (green color) and reference strains (black color, bold font).

**Figure 2:**
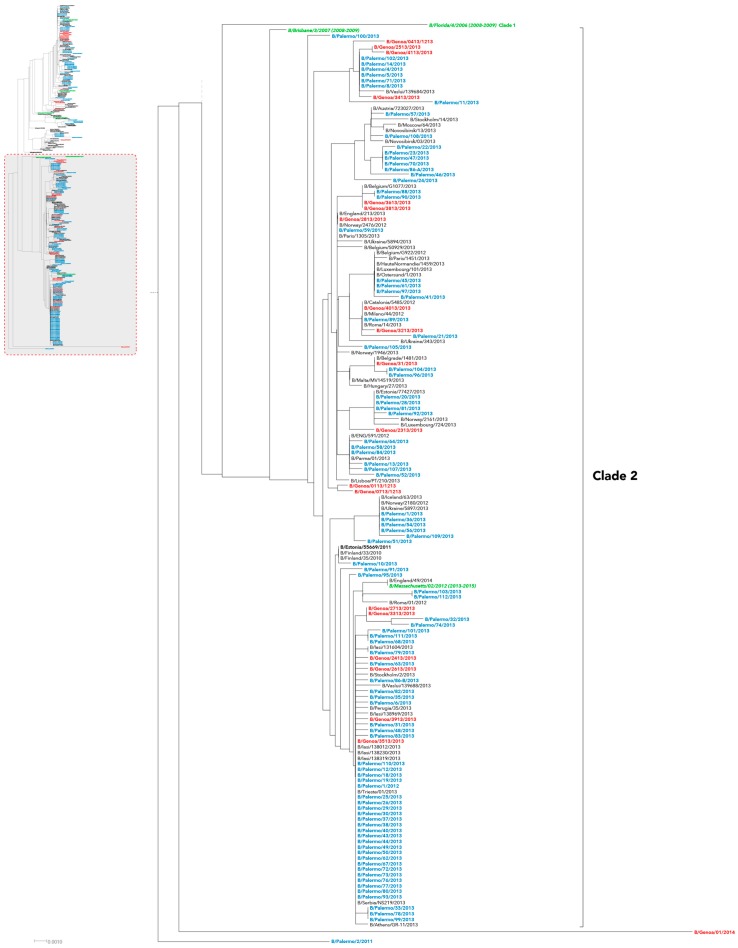
Phylogenetic analysis of the HA nucleotide sequences from influenza B Yamagata-lineage clade 2 viruses isolated in Italy from 2010 to 2015. The phylogeny tree was generated by the neighbor-joining method with 1000 bootstrap replicates. Sicilian strains (blue color), Ligurian strains (red color), WHO recommended candidate vaccine (green color) and reference strains (black color, bold font).

**Figure 3:**
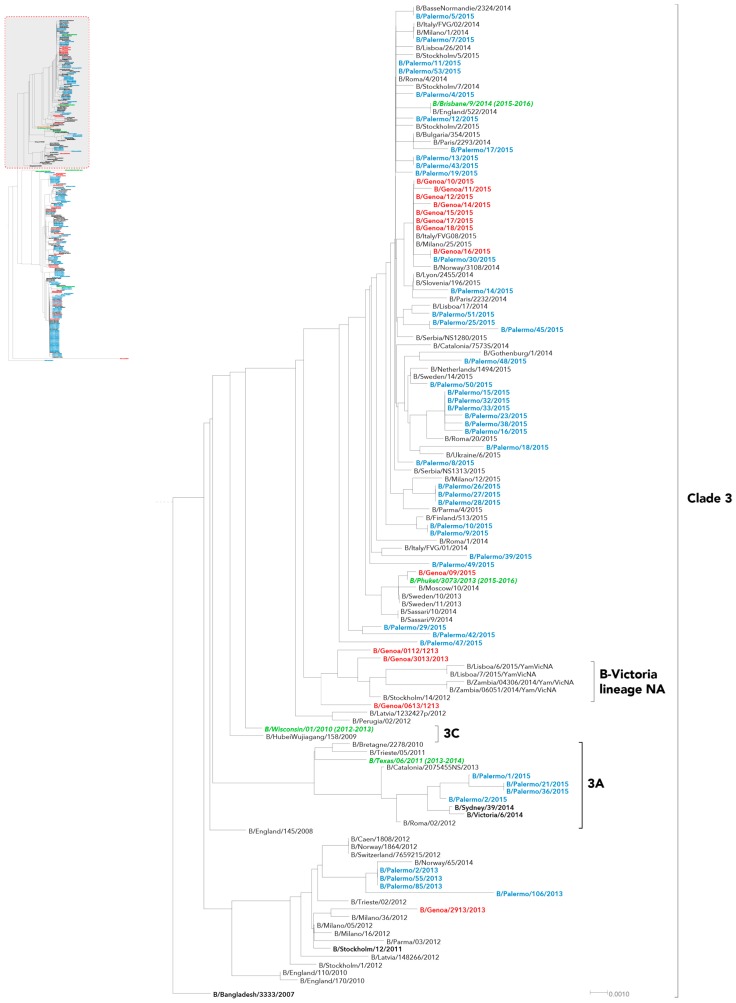
Phylogenetic analysis of the HA nucleotide sequences from influenza B Yamagata-lineage clade 3 viruses isolated in Italy from 2010 to 2015. The phylogeny tree was generated by the neighbor-joining method with 1000 bootstrap replicates. Sicilian strains (blue color), Ligurian strains (red color), WHO recommended candidate vaccine (green color) and reference strains (black color, bold font).

In our series, evaluation of HA phylogeny identified three different genetic groups for influenza B/Victoria-like strains (Figure 1) and two other major genetic groups for the B/Yamagata-like strains (Figure 2 and Figure 3), which appeared time-related and, consequently, revealing that there had been different independent introduction of the two influenza B lineages into Italy.

Most of B/Victoria-lineage viruses fell into clade 1 (Figure 1). In particular, influenza B viruses circulating in Sicily during the 2012–2013 season clustered in the subclade 1A with B/Brisbane/60/2008, whereas the Sicilian viruses obtained two influenza outbreaks earlier (season 2010–2011) grouped in the subclade 1B, which is represented by B/Hong Kong/259/2010–B/Bangladesh/5945/2009.

A different pattern was depicted through the analysis of clusters for influenza B Victoria-like viruses detected in Liguria, which did not show a chronological correlation with a specific surveillance season and similarly allocated within the two Victoria subclades.

Additionally, four strains sharing two small clusters of sequences, the first one including B/Enna/2/2011 and B/Palermo/4/2011 and the second one consisting of B/Palermo/4/2011 and B/Genoa/19/2015, were not clearly assigned to a specific Victoria clade. The two clusters showed 96.5% and 93.1% intragroup nucleotide similarity, respectively.

Overall, B/Yamagata-lineage Italian strains clustered into two distinguishable genetic clades: clade 2 and clade 3. Almost all B strains detected in 2013 were classified as B/Yamagata clade 2 (Figure 2), whereas only four HA sequences from Liguria and as much from Sicily fell into clade 3 (Figure 3). These latter ones formed a monophyletic group with other Italian and European viruses identified in 2012.

Conversely, all of the strains recently identified between 2014 and 2015 grouped into clade 3, with a small set of viral sequences from Sicily which were distinguishable from the great majority of them, sharing the same cluster within the genetic clade 3A (Figure 3).

Finally, one HA sequence from Sicily (B/Palermo/2/2011) and one from Liguria (B/Genoa/01/2014) substantially diverged from the other B/Yamagata strains (Figure 2).

Based on HA phylogeny, the overall prevalence of strains belonging to B/Victoria clade 1, B/Yamagata clade 2, and B/Yamagata clade 3 were 14.7% (*n* = 29/197), 56.8% (*n* = 112/197), and 28.4% (*n* = 56/197), respectively.

**Figure 4:**
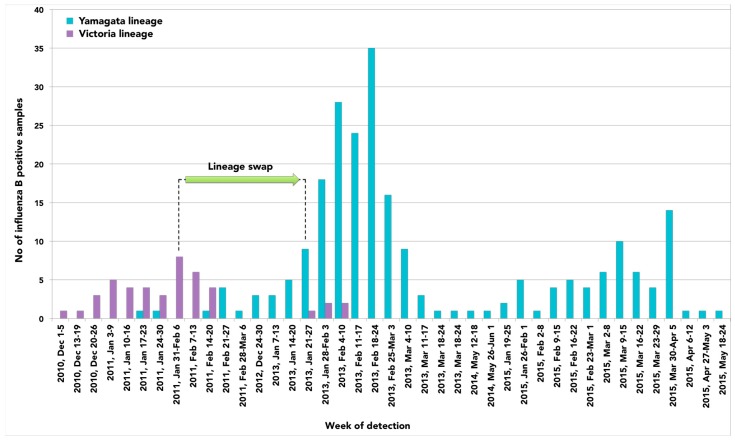
Lineage swap of influenza B viruses occurred in Italy between 2010 and 2015.

In general, considering the number of influenza B positive samples collected over time and the lack of influenza B detection in 2012, our findings suggest that a lineage swap occurred in Italy between January 2011 and January 2013 (Figure 4). Although co-circulation of B/Yamagata-lineage and B/Victoria-lineage was observed, the latter one predominated in the season 2010–2011 followed by B/Yamagata-like strains in the season 2012–2013 that remained since then.

The deduced HA amino-acid sequences of the influenza B strains included in this study were also evaluated.

On average, all Italian B/Victoria clade 1, B/Yamagata clade 2 and B/Yamagata clade 3 strains showed more than 98% amino-acid similarity with the corresponding representative vaccine strain (B/Brisbane/60/2008 for Victoria clade 1, B/Massachussets/02/2010 for Yamagata clade 2, and B/Wisconsin /02/2010 for Yamagata clade 3).

According to B lineage and clade [19,20], several deduced amino-acid substitutions on the HA protein were detected and some occurred on selected sites of the four major epitopes of HA1 coding region of influenza B viruses from Liguria and Sicily (Table 1).

In details, the most represented substitutions were N116K in the 120-loop, shared by 83.9% (*n* = 47/56) of B/Yamagata clade 3 strains and I146V in the 150-loop, which was detected in 89.6% (*n* = 26/29) of B/Victoria clade 1 strains. D197N and D196N in 190-helix (based on Victoria clade 1 and Yamagata clade 2/Yamagata clade 3 vaccine strains numbering, respectively), which represent key residues at the glycosylation site, were found in almost all viruses collected in the two Italian regions during the study period (100%, *n* = 29/29; 100%, *n* = 112/112; 96.4%, *n* = 54/56, for B/Victoria clade 1, B/Yamagata clade 2 and B/Yamagata clade 3, respectively).

Interestingly, substitutions H122Q and N126D for B/Victoria viruses, and K197R and S202N for B/Yamagata clade 3 viruses were exclusively observed in a few Sicilian strains, while substitution N126S in the 120-loop was recorded in only one isolate from Liguria.

Table 2 and Table 3 report the serological characterization of selected Ligurian isolates and reference viruses (B/Brisbane/60/2008 for Victoria-lineage viruses and B/Wisconsin/01/2010, B/Massachusetts/02/2012 and B/Phuket/3073/2013 for Yamagata-lineage viruses) and the amino-acid mutations with respect to B/Brisbane/60/2008 and B/Wisconsin/01/2010, respectively.

As for B/Victoria isolates (Table 2), B/Genoa/22/2011 showed the closest relationship with the vaccine strain B/Brisbane/60/2008 in terms of hemagglutination inhibition (HI) titer, although the predicted amino-acid sequences of the HA1 domains of the strains showed two differences in the sequenced region (I37V and G80R). B/Genoa/04/2010 had only one amino-acid substitution with respect to B/Brisbane/60/2008 (V146I), but this is located in the 150-loop, one of the most important antibody-binding sites on influenza B virus HA protein: this single mutation affected the HI titration, showing a quite different antigenic profile between the clinical isolate and the vaccine strain, although they belonged to the same phylogenetic clade (1A).

The same mutation was observed in B/Genoa/08/2010 and B/Genoa/12/2011 viruses, phylogenetically belonging to B/Victoria clade 1B: together with P58L substitution, that fell within 120-loop, these two mutations heavily affected the antigenic pattern of the Ligurian viruses, as also shown by the low HI titer.

As regards B/Yamagata isolates (Table 3), selected Ligurian viruses presented a number of amino-acid mutations onto the four major influenza B HA epitopes, with respect to reference vaccine strains, that affected HI titration as well as the antigenic profile of the viruses.

B/Genoa/0413/1213 and B/Genoa/0713/1213 phylogenetically belonged to B/Yamagata clade 2, the same clade of the reference strain B/Massachusetts/02/2012, and they showed the same amino-acid substitutions, in particular a lysine (K) residue at position 48, a serine (S) at position 150, a threonine (T) at position 199, and an asparagine (N) at positions 166 and 203. This similarity was partially reflected by HI assay: the two clinical isolates and the reference strain showed analogous titers when tested against B/Wisconsin/01/2010 and B/Phuket/3073/2013 post infection ferret antisera, although the Ligurian viruses presented low titers when tested with B/Massachusetts/02/2012 antisera.

Among tested Ligurian isolates belonging to B/Yamagata clade 3, B/Genoa/0112/1213 and B/Genoa/0613/1213 presented a close relationship with the reference strain B/Phuket/3073/2013 at HI assay, with which they shared the same amino-acid substitutions K116N in the 120-loop and T199I in the 190-helix. The two selected Ligurian viruses isolated in 2015 showed a different antigenic pattern, although the quite similar distribution of amino-acid substitutions and the low phylogenetic distance. B/Genoa/11/2015 presented the highest HI titers towards all three reference vaccine strains and only two amino-acid substitutions in the four major epitopes with respect to B/Wisconsin/01/2010, namely K116N in the 120-loop and T199I in the 190-helix. Conversely, B/Genoa/09/2015 showed reduced HI titers when tested with B/Wisconsin/01/2010 and B/Massachusetts/02/2012 antisera, although it presented only two differences with B/Genoa/11/2015 in the entire sequenced region: noteworthy, B/Genoa/09/2015 presented an aspartic acid (D) at position 197, a key residue at the glycosylation site.

B/Genoa/01/2014 phylogenetically diverged from the other B/Yamagata strains and showed distinguished antigenic and molecular patterns: in particular, this isolate was characterized by the lowest HI titers towards the three reference strains and several amino-acid substitutions within and across the major epitopes of influenza B HA protein.

## 3. Discussion

Analysis of nucleotide sequences of the HA gene revealed different introduction of B viruses in the two administrative regions of Italy considered and confirming the co-circulation of the two influenza B lineages.

Overall, B/Victoria-lineage strains identified in Sicily and Liguria throughout the study period belonged to the phylogenetic clade 1. While all influenza B viruses obtained in 2013 exclusively clustered within subclade 1A [21], strains from previous seasons similarly distributed among subclades 1A and 1B, but showing some level of genetic heterogeneity as highlighted by the two small group of HA sequences, which fell outside clade 1 and represented by B/Palermo/4/2011 and B/Palermo/8/2011.

A different picture was observed for Yamagata-lineage viruses that predominated over Victoria-lineage strains since 2012–2013. Noteworthy, this lineage shift coincided with a significant and increased proportion of influenza B viruses circulating in our country.

B/Yamagata-lineage clade 2 harbored most of HA sequences of viruses collected during the earlier seasons, whereas only four grouped within genetic clade 3, in the same cluster of both the reference strain B/Stockholm/12/2011 and other European strains obtained from 2012 to 2014, similarly retracing the European picture as documented by European Centre for Disease Prevention and Control (ECDC) in the middle 2013 [22].

On the other hand, the great majority of B/Yamagata-lineage sequences obtained during the last season 2014–2015 fell into the genetic clade 3, recalling the worldwide scenario over the same time period [23,24,25].

Finally, a small proportion of Yamagata viruses collected in Palermo clustered within a group antigenically dissimilar to the other viruses of clade 3, designated as clade 3A and represented by some Australian strains collected in late 2014 and sporadically documented elsewhere in the world [23].

The mechanism that drives the turnover of distinct B lineages is still not clear, but it could reasonably be a result of immune selection due to accumulated herd immunity in the human population [26]. In this regard, the prevalent circulation of B/Victoria-lineage strains documented in Europe by ECDC during the current season 2015–2016 [27], as also confirmed using preliminary molecular data available in Sicily and Liguria as part of the active virologic surveillance system, overturns the predominance of B/Yamagata-lineage viruses found during the previous season 2014–2015.

The hemagglutinin, as one of the two major surface glycoproteins of influenza virus, represents a primary target for host neutralizing antibodies. More specifically, the HA1 subunit contains the receptor-binding sites and the most part of antigenic sites, among which four major epitopes named 120-loop, 150-loop, 160-loop, and 190-helix [28] have been demonstrated to undergo positive selective pressure in the course of evolution [19,29].

Irrespective to their lineage, all influenza viruses considered in this study showed identity at residues Phe-95, Trp-158, His-191 and Tyr-202, four amino-acids which form the base of the receptor-binding site on the HA protein and which appear to be absolutely conserved among all known influenza B viruses [20].

Nevertheless, several amino-acid substitutions were found on the four major HA epitopes of the viruses detected in the two Italian regions, as compared to their specific lineage reference strains. These mutations were mainly observed on the 120-loop and the 190-helix, while the other two antigenic sites demonstrated a lower level of variability, either among Victoria-lineage or Yamagata-lineage strains, especially the 150-loop.

It is well known that the residues located on the antigenic determinant 120-loop play a very important role in stabilizing the structure of hemagglutinin protein, providing protection from being hydrolyzed and, consequently, evading recognition by neutralizing antibodies.

In our setting, the most frequent deduced amino-acid substitutions were N116K in the 120-loop of B/Yamagata clade 3 strains and I146V in the 150-loop of Victoria strains; the latter one shared by none of the Yamagata-lineage viruses. Of note, all viruses analyzed in this study presented a N-linked glycosylation site at the residue 196/197 of the 190-helix, while a new glycosilation residue was observed at the position 202 in the 190-helix of about 20% Yamagata clade 3 influenza B strains identified in Sicily only, suggesting different geographic patterns of evolution in our country, with respect to this mutation.

Selected Ligurian viruses for antigenic characterization represented almost all the phylogenetic clades that harbored influenza B sequences collected in the period 2010–2015.

Hemagglutination inhibition assays allow to estimate the antigenic distance between the vaccine candidates and the circulating strains of influenza virus: our results showed a heterogeneous pattern among Ligurian isolates, with co-circulation of influenza B viruses closely related to the vaccine strains and viruses presenting lower reactivity with the reference serum. As far as B/Victoria lineage, viruses in clade 1A were antigenically distinguishable from those in clade 1B; noteworthy, although belonging to clade 1A, B/Genoa/04/2010 showed a four-fold reduction in HI titer in the presence of vaccine antisera, suggesting a sub-optimal match with the vaccine candidate. As regards B/Yamagata strains, viruses isolated during 2012–2013 presented low HI titers against vaccine candidate B/Wisconsin/01/2010 and B/Massachusetts/02/2012, regardless of clade, while a closer relationship between isolates in clade 3 and B/Phuket/3073/2013 emerged. Tested viruses isolated in 2015 showed a good match with the vaccine strain B/Phuket/3073/2013, with HI titers within two-fold of that of the homologous virus.

Interestingly, B/Genoa/01/2014, the only 2014 isolates available for analysis, was not clearly assigned to a recognized B/Yamagata clade, showed several amino-acid substitutions on the HA1 coding region with respect to vaccine candidates and presented very low HI titer against reference antisera.

The effects of these changes on the antigenic properties of the influenza virus, on the seroprotection and on-field effectiveness of available influenza vaccine are poorly understood. The HI test allows to reveal the major changes in antigenicity that are observed when a drift occurs, but isolates with few amino-acid changes over antigenic sites are not clearly discriminated. It is evident, therefore, that combined serologic and molecular analyses are necessary to better evaluate the characteristics of any new or re-emerging influenza B viruses, in order to achieve the best match between vaccine and circulating strains.

In the light of molecular characterization of HA, antigenic analysis confirmed the high variability of circulating influenza B virus, although some observed antigenic patterns cannot be clearly explained, confirming the difficulty in correlating the antigenic data with sequence analysis, as already observed in other experiences, also involving influenza A viruses [30,31,32].

In summary, this paper highlights the importance of continuous molecular surveillance of influenza B viruses in order to better understand the genetic dynamics of these viruses and to monitor the variation of epidemiology in Italy.

Although the lack of available data from other influenza virus gene segments may represent an important limitation of this study, the results presented here provide evidence of heterogeneity and new molecular reference for estimating the variability of circulating influenza B viruses in our geographic area.

Based on epidemiological data collected from the two Italian regions during the last five-year time period, a significant degree of mismatch of trivalent seasonal vaccines has been observed in respect to circulating influenza B virus strains (Tramuto, F.; personal communication), and it unquestionably represents a challenge in terms of efficacy and effectiveness of vaccine formulations currently used.

In conclusion, our results confirm the substantial unpredictability of seasonal proportion of different influenza types and lineages. Because of the limited cross-protection between B viruses lineages, the use of influenza vaccines based on the trivalent formulation could provide inadequate immunization to the entire population against influenza B infection, adding further evidence to support the adoption of quadrivalent vaccines in universal vaccination programs.

## 4. Materials and Methods

### 4.1. Study Population and Clinical Specimens Collection

We analyzed virological data obtained from influenza B positive nasopharyngeal samples collected in Sicily and Liguria from subjects presenting ILI symptoms [33] between 2010 and 2015, as part of the nationwide influenza surveillance network implemented in Italy [13,34]. Influenza strains were labeled according to the internationally accepted naming convention [35].

Nasopharyngeal samples were collected and transported to the regional reference laboratories by using Virocult swabs (MWE, Medical Wire, Corsham, UK).

Total nucleic acids were extracted using QIAamp Viral RNA extraction kit (QIAGEN, Hilden, Germany) according to the manufacturer’s suggested protocol, eluted from the spin column in 60 μL of elution buffer, divided into aliquots and stored at −80 °C until further use. Each sample was routinely tested by one-step real-time RT-PCR for the presence of influenza virus RNA, and influenza B positive samples were genotyped using lineage-specific multiplex one-step real-time RT-PCR according the “WHO protocols for molecular diagnosis of influenza virus” [36] using a QuantStudio 7 Flex Real-Time PCR system (Applied Biosystems, Carlsbad, CA, USA).

A representative subset of influenza B positive specimens was further genetically characterized by direct sequencing of HA gene. Sequencing approach involved the design of three primer sets, which amplify overlapping fragments that span the entire gene’s sequence (Supplementary Figure S2).

The sequencing PCR was performed using the Superscript^®^ III One-Step RT-PCR System with Platinum^®^ Taq DNA polymerase (Life Technologies, Carlsbad, CA, USA). Cycling condition included one cycle of reverse transcription at 50 °C for 30 min; one cycle of initial PCR activation at 94 °C for 2 min; 45 cycles of denaturation at 94 °C for 15 s, primer annealing at 55 °C for 30 s, and extension at 68 °C for 1.5 min; the reaction ended with an extension step at 68 °C for 8 min to assure completion of partial double-stranded molecules.

Purified PCR products were directly sequenced by cycle sequencing using Big Dye Terminator chemistry v3.1 on an ABI Prism 3130xl automatic sequencer.

### 4.2. Phylogenetic Analysis and Antigenic Characterization

All HA gene sequence data were edited and assembled using CLC Main Workbench software package v7.6.4. For phylogenetic analysis, a set of HA nucleotide sequences of the WHO recommended vaccine and other representative influenza B strains, collected during the selected study period, were obtained from both the databases of the Global Initiative on Sharing All Influenza Data (GISAID) [37] and the National Center for Biotechnology Information (NCBI Influenza Virus Resource [38].

Multiple sequence alignment with the reference strains was performed with ClustalX v2.0 [39], nucleotide/amino-acids alignments were manually edited with BioEdit [40], and phylogenetic trees were generated using MEGA v6.06 software [41] applying the neighbor-joining method with 1000 bootstrap replicates.

The panel of HA nucleotide sequences of the influenza B strains used in this paper included all available data from Sicily and Liguria in public databases at the time of writing (the designations, collection dates, GenBank accession numbers and relevant details are shown in Supplementary Table S1).

Antigenic characterization of a representative subset of influenza B viruses identified in Liguria during the study period was performed by hemagglutination inhibition (HI) test, using whole viruses and post-infection ferret sera to reference viruses (provided by WHO Influenza Collaborating Centre, London, UK), as previously described [13]. The Ligurian influenza B virus was isolated directly from the clinical samples grown in Madin-Darby canine kidney (MDCK-London) cell line.

## Figures and Tables

**Table 1:** Amino-acid substitutions found on the hemagglutinin (HA) protein of influenza B viruses detected in Italy during the period 2010–2015, according to B lineage.

Subunit	Epitope (Residue Location)	B/Victoria	B/Yamagata	
		Clade 1 (*n *= 29)	Clade 2 (*n *= 112)	Clade 3 (*n *= 56)
**HA1**	120-loop (116–137)	H116N (2)		
		T121S (1)		
		H122Q (2) *	N116K (1)	N116K (47)
		N126D (1) *	S120T (2)	Q122K (4)
		N126S (1) **	T121S (9)	N123T (4)
		E128A (1)	D126N (1)	D126N (1)
		N129K (2)		
		N129D (4)		
	150-loop (141–150)	I146V (26)	T139A (1)	I150V (3)
			S150I (3)	I150S (1)
	160-loop (162–167)	N163D (1)	D163N (1) N165Y (3)	
		D164N (1)		
		K165N (1)		-
		K167R (1)		
		K167N (1)		
	190-helix (194–202)	D197N (29) E198K (1) A202Q (1) A202K (1)	D196N (112)	D196N (54)
			K197E (1)	D196Y (1)
			K201A (1)	K197R (3) *
			N202K (1)	T198S (1)
			N202S (2)	S202N (9) *

Amino-acid substitutions are defined according to vaccine strains of respective clades and are numbered according to respective vaccine strains (B/Brisbane/60/2008 for B/Victoria clade 1, B/Massachussetts/02/2012 for B/Yamagata clade 2, and B/Wisconsin/01/2010 for B/Yamagata clade 3). The number of strains having the substitution is indicated inside parenthesis. * Deduced amino-acid substitutions detected in Sicilian isolates only. ** Deduced amino-acid substitutions detected in Ligurian isolates only.

**Table 2:** Antigenic relationships between selected Ligurian Influenza B isolates and B/Brisbane/60/2008 Victoria-lineage reference virus and the deduced amino-acid mutations found on the HA1 coding region with respect to B/Brisbane/60/2008 (reference viruses, homologous titers and epitopes are in bold type).

**Influenza B Victoria-lineage Virus (Clade)**	**HI Titer Post Infection Ferret Antisera B/Brisbane/60/2008 F22/12 1A**	**Amino-Acid Mutations with Respect to B/Brisbane/60/2008**			
		**120-loop**			**150-loop**
		37	**58**	80	**146**
**B/Brisbane/60/2008 (1A)**	**1280**	V	L	R	I
B/Genoa/04/2010 (1A)	320	-	-	-	V
B/Genoa/08/2010 (1B)	160	-	P	-	V
B/Genoa/12/2011 (1B)	160	-	P	-	V
B/Genoa/22/2011 (1A)	640	I	-	G	-

V, Valine; L, Leucine; R, Arginine; I, Isoleucine; P, Proline; G, Glycine.

**Table 3:** Antigenic relationships between selected Ligurian Influenza B isolates and B/Wisconsin/01/2010, B/Massachusetts/02/2012 and B/Phuket/3073/2013 Yamagata-lineage reference viruses and the deduced amino-acid mutations found on the HA1 coding region with respect to B/Wisconsin/01/2010 (reference viruses, homologous titers and epitopes are in bold type).

**Influenza B Yamagata-Lineage Virus (Clade)**	**HI Titer Post Infection Ferret Antisera**			**Amino-Acid Mutations with Respect to B/Wisconsin/01/2010**																							
	**B/Wisc/ 01/10**	**B/Mass/ 02/12**	**B/Phuk/ 3073/13**	**120-loop**				**150-loop**	**160-loop**						**190-helix**												
				47	**48**	108	**116**	**150**	**165**	**166**	**168**	173	175	182	**197**	**198**	**199**	**202**	**203**	230	233	252	255	262	267	299	313
**B/Wisc/01/10 (3C)**	**1280**	320	160	T	R	P	N	I	N	Y	N	L	V	T	N	K	I	K	S	D	D	M	P	V	V	K	E
**B/Mass/02/12 (2)**	1280	**640**	320	-	K	A	-	S	-	N	-	-	-	A	-	-	T	-	N	-	-	-	-	-	-	-	-
**B/Phuk/3073/13 (3)**	640	320	**1280**	-	-	-	K	-	-	-	-	-	-	-	-	-	T	-	-	-	-	-	-	-	-	E	K
B/Genoa/0112/1213 (3)	320	160	1280	A	-	-	K	V	-	-	-	-	-	-	-	-	T	-	-	-	-	-	-	-	-	E	K
B/Genoa/0413/1213 (2)	640	160	160	-	K	A	-	S	-	N	-	-	-	A	-	-	T	-	N	G	-	-	-	-	-	-	-
B/Genoa/0613/1213 (3)	160	160	640	A	-	-	K	S	-	-	-	-	-	-	-	-	T	-	-	-	-	-	-	-	-	E	K
B/Genoa/0713/2013 (2)	640	160	80	-	K	A	-	S	-	N	-	-	-	A	-	-	T	-	N	G	-	-	-	-	-	-	-
B/Genoa/01/2014 (nd)	160	80	80	-	-	-	K	-	K	-	T	Q	I	-	-	E	T	A	K	G	N	V	S	T	I	-	-
B/Genoa/09/2015 (3)	80	80	640	-	-	-	K	-	-	-	-	-	-	-	D	-	T	-	-	-	-	-	-	-	-	E	K
B/Genoa/11/2015 (3)	1280	320	2560	-	-	-	K	-	-	-	-	Q	-	-	-	-	T	-	-	-	-	-	-	-	-	E	K

nd: not determined. T, Threonine; R, Arginine ; P, Proline; N, Asparagine; I, Isoleucine; Y, Tyrosine; L, Leucine; V, Valine; K, Lysine; S, Serine; D, Aspartic Acid; M, Methionine; E, Glutamic Acid; A, Alanine; G, Glycine; Q, Glutamine.

## Supplementary Materials

Supplementary materials can be found at http://www.mdpi.com/1422-0067/17/4/549/s1.

## Abbreviations

HA: Hemagglutinin
ILI: Influenza-like illness

## References

[1] Fraaij P.L., Heikkinen T. (2001). Seasonal influenza: The burden of disease in children. Vaccine.

[2] Paul Glezen W., Schmier J.K., Kuehn C.M., Ryan K.J., Oxford J. (2013). The burden of influenza B: A structured literature review. Am. J. Public Health.

[3] Reed C., Chaves S.S., Daily Kirley P., Emerson R., Aragon D., Hancock E.B., Butler L., Baumbach J., Hollick G., Bennett N.M. (2015). Estimating influenza disease burden from population-based surveillance data in the United States. PLoS ONE.

[4] Osterhaus A.D., Rimmelzwaan G.F., Martina B.E., Bestebroer T.M., Fouchier R.A. (2000). Influenza B virus in seals. Science.

[5] Bodewes R., Morick D., de Mutsert G., Osinga N., Bestebroer T., van der Vliet S., Smits S.L., Kuiken T., Rimmelzwaan G.F., Fouchier R.A. (2013). Recurring influenza B virus infections in seals. Emerg. Infect. Dis..

[6] Ran Z., Shen H., Lang Y., Kolb E.A., Turan N., Zhu L., Ma J., Bawa B., Liu Q., Liu H. (2015). Domestic pigs are susceptible to infection with influenza B viruses. J. Virol..

[7] Matsuzaki Y., Sugawara K., Takashita E., Muraki Y., Hongo S., Katsushima N., Mizuta K., Nishimura H. (2004). Genetic diversity of influenza B virus: The frequent reassortment and cocirculation of the genetically distinct reassortant viruses in a community. J. Méd. Virol..

[8] Nerome R., Hiromoto Y., Sugita S., Tanabe N., Ishida M., Matsumoto M., Lindstrom S.E., Takahashi T., Nerome K. (1998). Evolutionary characteristics of influenza B virus since its first isolation in 1940: Dynamic circulation of deletion and insertion mechanism. Arch. Virol..

[9] Kanegae Y., Sugita S., Endo A., Ishida M., Senya S., Osako K., Nerome K., Oya A. (1990). Evolutionary pattern of the hemagglutinin gene of influenza B viruses isolated in Japan: Cocirculating lineages in the same epidemic season. J. Virol..

[10] Lin Y.P., Gregory V., Bennett M., Hay A. (2004). Recent changes among human influenza viruses. Virus Res..

[11] Paiva T.M., Benega M.A., Silva D.B., Santos K.C., Cruz A.S., Hortenci M.F., Barbieri M.T., Monteiro M.M., Barbosa H.A., Carvalhanas T.R. (2013). Evolutionary pattern of reemerging influenza B/Victoria-lineage viruses in São Paulo, Brazil, 1996–2012: Implications for vaccine composition strategy. J. Med. Virol..

[12] Caini S., Huang Q.S., Ciblak M.A., Kusznierz G., Owen R., Wangchuk S., Henriques C.M., Njouom R., Fasce R.A., Yu H. (2015). Global influenza B study, epidemiological and virological characteristics of influenza B: Results of the global influenza B study. Influenza Other Respir. Viruses.

[13] Ansaldi F., D'Agaro P., de Florentiis D., Puzelli S., Lin Y.P., Gregory V., Bennett M., Donatelli I., Gasparini R., Crovari P. (2003). Molecular characterization of influenza B viruses circulating in northern Italy during the 2001–2002 epidemic season. J. Med. Virol..

[14] Puzelli S., Frezza F., Fabiani C., Ansaldi F., Campitelli L., Lin Y.P., Gregory V., Bennett M., D'Agaro P., Campello C. (2004). Changes in the hemagglutinins and neuraminidases of human influenza B viruses isolated in Italy during the 2001–2002, 2002–2003, and 2003–2004 seasons. J. Med. Virol..

[15] Skowronski D.M., Janjua N.Z., Sabaiduc S., de Serres G., Winter A.L., Gubbay J.B., Dickinson J.A., Fonseca K., Charest H., Bastien N. (2014). Influenza A/subtype and B/lineage effectiveness estimates for the 2011–2012 trivalent vaccine: Cross-season and cross-lineage protection with unchanged vaccine. J. Infect. Dis..

[16] Lo Y.C., Chuang J.H., Kuo H.W., Huang W.T., Hsu Y.F., Liu M.T., Chen C.H., Huang H.H., Chang C.H., Chou J.H. (2013). Surveillance and vaccine effectiveness of an influenza epidemic predominated by vaccine-mismatched influenza B/Yamagata-lineage viruses in Taiwan, 2011–2012 season. PLoS ONE.

[17] Ambrose C.S., Levin M.J. (2012). The rationale for quadrivalent influenza vaccines. Hum. Vaccines Immunother..

[18] Heikkinen T., Ikonen N., Ziegler T. (2014). Impact of influenza B lineage-level mismatch between trivalent seasonal influenza vaccines and circulating viruses, 1999–2012. Clin. Infect. Dis..

[19] Wang Q., Cheng F., Lu M., Tian X., Ma J. (2008). Crystal structure of unliganded influenza B virus hemagglutinin. J. Virol..

[20] Ni F., Kondrashkina E., Wang Q. (2013). Structural basis for the divergent evolution of influenza B virus hemagglutinin. Virology.

[21] European Centre for Disease Prevention and Control (2013). Influenza Virus Characterisation, Summary Europe. http://ecdc.europa.eu/en/publications/Publications/influenza-virus-characterisation-mar-2013.pdf.

[22] European Centre for Disease Prevention and Control (2013). Influenza Virus Characterisation, Summary Europe. http://ecdc.europa.eu/en/publications/Publications/influenza-virus-characterisation-june-2013.pdf.

[23] European Centre for Disease Prevention and Control (2015). Influenza Virus Characterisation, Summary Europe. http://ecdc.europa.eu/en/publications/Publications/influenza-virus-characterisation-june-2015.pdf.

[24] Oong X.Y., Ng K.T., Lam T.T., Pang Y.K., Chan K.G., Hanafi N.S., Kamarulzaman A., Tee K.K. (2015). Epidemiological and Evolutionary Dynamics of Influenza B Viruses in Malaysia, 2012–2014. PLoS ONE.

[25] Tewawong N., Suwannakarn K., Prachayangprecha S., Korkong S., Vichiwattana P., Vongpunsawad S., Poovorawan Y. (2015). Molecular epidemiology and phylogenetic analyses of influenza B virus in Thailand during 2010 to 2014. PLoS ONE.

[26] Chen R., Holmes E.C. (2008). The evolutionary dynamics of human influenza B virus. J. Mol. Evol..

[27] European Centre for Disease Prevention and Control Influenza Surveillance Data. http://ecdc.europa.eu/en/healthtopics/seasonal_influenza/epidemiological_data/Pages/Latest_surveillance_data.aspx.

[28] Knossow M., Skehel J.J. (2006). Variation and infectivity neutralization in influenza. Immunology.

[29] Shen J., Kirk B.D., Ma J., Wang Q. (2009). Diversifying selective pressure on influenza B virus hemagglutinin. J. Med. Virol..

[30] Hay A.J., Lin Y.P., Gregory V., Benett M. (2001). Annual Report—WHO Influenza Centre.

[31] Hay A.J., Lin Y.P., Gregory V., Benett M. (2002). Annual Report—WHO Influenza Centre.

[32] Ansaldi F., Bacilieri S., Amicizia D., Valle L., Banfi F., Durando P., Sticchi L., Gasparini R., Icardi G., Crovari P. (2004). Antigenic characterisation of influenza B virus with a new microneutralisation assay: Comparison to haemagglutination and sequence analysis. J. Med. Virol..

[33] Ministero della Salute—Repubblica Italiana InfluNet: Protocollo Operativo—Stagione Influenzale 2015–2016. http://www.salute.gov.it/imgs/C_17_pubblicazioni_2418_allegato.pdf.

[34] Tramuto F., Maida C.M., Bonura F., Perna A.M., Puzelli S., de Marco M.A., Donatelli I., Aprea L., Firenze A., Arcadipane A. (2011). Surveillance of hospitalised patients with influenza-like illness during pandemic influenza A(H1N1) season in Sicily, April 2009–December 2010. Eur. Surveill..

[35] World Health Organization (1980). A revision of the system of nomenclature for influenza viruses: A WHO memorandum. Bull. World Health Organ..

[36] World Health Organization (2014). WHO Information for Molecular Diagnosis of Influenza Virus. http://www.who.int/entity/influenza/gisrs_laboratory/molecular_diagnosis_influenza_virus_humans_update_201403rev201505.pdf?ua=1.

[37] Global Initiative on Sharing All Influenza Data. http://platform.gisaid.org.

[38] National Center for Biotechnology Information. http://www.ncbi.nlm.nih.gov/genomes/FLU/FLU.html.

[39] Larkin M.A., Blackshields G., Brown N.P., Chenna R., McGettigan P.A., McWilliam H., Valentin F., Wallace I.M., Wilm A., Lopez R. (2007). Clustal W and Clustal X version 2.0. Bioinformatics.

[40] Hall T. (2011). BioEdit: An important software for molecular biology. GERF Bull. Biosci..

[41] Tamura K., Stecher G., Peterson D., Filipski A., Kumar S. (2013). MEGA6: Molecular evolutionary genetics analysis version 6.0. Mol. Biol. Evol..

