# Supplementary material for: The Molecular Epidemiology and Evolutionary Dynamics of Influenza B Virus in Two Italian Regions during 2010–2015: The Experience of Sicily and Liguria
Source: Int J Mol Sci. 2016 Apr 13;17(4):549. doi: 10.3390/ijms17040549 (PMC4849005; doi:10.3390/ijms17040549)
Supplement: Supplementary file 1 [file ijms-17-00549-s001.pdf]

Supplementary Materials: The Molecular Epidemiology and Evolutionary Dynamics of Influenza B Virus in Two Italian Regions during 2010–2015: The Experience of Sicily and Liguria

Fabio Tramuto, Andrea Orsi, Carmelo Massimo Maida, Claudio Costantino, Cecilia Trucchi, Cristiano Alicino, Francesco Vitale and Filippo Ansaldi

Table S1. Relevant details of Influenza B strains identified in Sicily and Liguria between 2010 and 2015.

GENBANK ACCESSION NUMBER	VIRUS NAME	B LINEAGE	B CLADE	COLLECTION DATE	AGE	SEX	SOURCE
Pending	B/Genoa/01/2009	Victoria-like	1A	NA	NA	NA	NA
Pending	B/Genoa/03/2009	Victoria-like	1A	NA	NA	NA	NA
Pending	B/Genoa/04/2010	Victoria-like	1A	20/12/2010	1 year	Male	Hospital
Pending	B/Genoa/02/2011	Victoria-like	1A	03/01/2011	11 years	Female	Hospital
Pending	B/Genoa/22/2011	Victoria-like	1A	18/01/2011	49 years	Male	Hospital
KU529067	B/Palermo/3/2013	Victoria-like	1A	21/01/2013	2 years	Female	Community
KU529065	B/Palermo/16/2013	Victoria-like	1A	31/01/2013	7 years	Male	Community
KU529066	B/Palermo/17/2013	Victoria-like	1A	31/01/2013	12 years	Female	Community
KU529085	B/Palermo/27/2013	Victoria-like	1A	05/02/2013	16 years	Female	Community
Pending	B/Genoa/02/2010	Victoria-like	1B	01/12/2010	5 years	Male	Hospital
Pending	B/Genoa/08/2010	Victoria-like	1B	30/12/2010	72 years	Male	Hospital
Pending	B/Genoa/12/2011	Victoria-like	1B	13/01/2011	65 years	Male	Hospital
KU529076	B/Palermo/1/2011	Victoria-like	1B	13/01/2011	42 years	Female	Community
KU529074	B/Palermo/5/2011	Victoria-like	1B	20/01/2011	19 years	Female	Community
KU529079	B/Palermo/6/2011	Victoria-like	1B	20/01/2011	16 years	Male	Community
KU529071	B/Palermo/7/2011	Victoria-like	1B	24/01/2011	15 years	Male	Community
KU529084	B/Palermo/9/2011	Victoria-like	1B	24/01/2011	16 years	Female	Community
KU529077	B/Palermo/10/2011	Victoria-like	1B	27/01/2011	47 years	Male	Community
KU529069	B/Enna/1/2011	Victoria-like	1B	29/01/2011	75 years	Male	Hospital
KU529070	B/Enna/3/2011	Victoria-like	1B	29/01/2011	31 years	Male	Hospital
KU529080	B/Palermo/11/2011	Victoria-like	1B	31/01/2011	50 years	Female	Community
KU529081	B/Palermo/12/2011	Victoria-like	1B	02/02/2011	20 years	Female	Hospital
KU529075	B/Palermo/13/2011	Victoria-like	1B	09/02/2011	11 years	Female	Hospital
KU529068	B/Messina/2/2011	Victoria-like	1B	11/02/2011	59 years	Female	Hospital
KU529078	B/Palermo/15/2011	Victoria-like	1B	12/02/2011	68 years	Male	Community
KU529083	B/Palermo/4/2011	Victoria-like	Non determined	20/01/2011	5 years	Male	Community
KU529073	B/Palermo/8/2011	Victoria-like	Non determined	24/01/2011	33 years	Female	Community
KU529082	B/Enna/2/2011	Victoria-like	Non determined	29/01/2011	63 years	Female	Hospital
Pending	B/Genoa/19/2015	Victoria-like	Non determined	23/05/2015	83 years	Female	Hospital
KU528915	B/Palermo/1/2012	Yamagata-like	2	31/12/2012	15 years	Male	Community
Pending	B/Genoa/0113/1213	Yamagata-like	2	07/01/2013	45 years	Male	Hospital
Pending	B/Genoa/2413/2013	Yamagata-like	2	07/01/2013	NA	NA	Hospital
KU528916	B/Palermo/1/2013	Yamagata-like	2	07/01/2013	7 years	Female	Community
Pending	B/Genoa/0413/1213	Yamagata-like	2	15/01/2013	68 years	Female	Hospital
Pending	B/Genoa/2313/2013	Yamagata-like	2	18/01/2013	NA	NA	Hospital
Pending	B/Genoa/2813/2013	Yamagata-like	2	18/01/2013	NA	NA	Hospital
Pending	B/Genoa/2513/2013	Yamagata-like	2	23/01/2013	NA	NA	Hospital
Pending	B/Genoa/2613/2013	Yamagata-like	2	23/01/2013	NA	NA	Hospital
Pending	B/Genoa/2713/2013	Yamagata-like	2	23/01/2013	NA	NA	Hospital
KU528918	B/Palermo/4/2013	Yamagata-like	2	24/01/2013	5 years	Female	Community
KU528919	B/Palermo/5/2013	Yamagata-like	2	24/01/2013	7 years	Male	Community
KU528920	B/Palermo/6/2013	Yamagata-like	2	25/01/2013	63 years	Male	Community
Pending	B/Genoa/31/2013	Yamagata-like	2	28/01/2013	NA	NA	Hospital
KU528922	B/Palermo/8/2013	Yamagata-like	2	28/01/2013	7 years	Female	Community
KU528923	B/Palermo/10/2013	Yamagata-like	2	28/01/2013	6 years	Male	Community
Pending	B/Genoa/0713/1213	Yamagata-like	2	29/01/2013	55 years	Male	Hospital
Pending	B/Genoa/3213/2013	Yamagata-like	2	29/01/2013	NA	NA	Hospital
Pending	B/Genoa/3313/2013	Yamagata-like	2	29/01/2013	NA	NA	Hospital

Table S1. *Cont.*

GENBANK ACCESSION NUMBER	VIRUS NAME	B LINEAGE	B CLADE	COLLECTION DATE	AGE	SEX	SOURCE
KU528926	B/Palermo/13/2013	Yamagata-like	2	29/01/2013	7 years	Female	Community
KU528927	B/Palermo/14/2013	Yamagata-like	2	29/01/2013	5 years	Male	Community
KU528924	B/Palermo/11/2013	Yamagata-like	2	29/01/2013	9 years	Female	Community
KU528925	B/Palermo/12/2013	Yamagata-like	2	29/01/2013	6 years	Male	Community
KU528928	B/Palermo/18/2013	Yamagata-like	2	31/01/2013	8 years	Male	Community
KU528929	B/Palermo/19/2013	Yamagata-like	2	31/01/2013	8 years	Female	Community
KU528930	B/Palermo/20/2013	Yamagata-like	2	01/02/2013	5 years	Female	Community
Pending	B/Genoa/3413/2013	Yamagata-like	2	02/02/2013	NA	NA	Hospital
Pending	B/Genoa/3513/2013	Yamagata-like	2	02/02/2013	NA	NA	Hospital
KU528931	B/Palermo/21/2013	Yamagata-like	2	04/02/2013	10 years	Female	Community
KU528932	B/Palermo/22/2013	Yamagata-like	2	04/02/2013	3 years	Male	Community
KU528933	B/Palermo/23/2013	Yamagata-like	2	04/02/2013	7 years	Female	Community
KU528934	B/Palermo/24/2013	Yamagata-like	2	04/02/2013	10 years	Female	Community
KU528936	B/Palermo/25/2013	Yamagata-like	2	04/02/2013	5 years	Female	Community
KU528935	B/Palermo/26/2013	Yamagata-like	2	05/02/2013	9 years	Male	Community
KU528937	B/Palermo/28/2013	Yamagata-like	2	05/02/2013	56 years	Female	Community
KU528938	B/Palermo/29/2013	Yamagata-like	2	05/02/2013	36 years	Female	Community
KU528939	B/Palermo/30/2013	Yamagata-like	2	05/02/2013	4 years	Male	Community
KU528940	B/Palermo/31/2013	Yamagata-like	2	05/02/2013	10 years	Female	Community
KU528941	B/Palermo/32/2013	Yamagata-like	2	05/02/2013	2 years	Male	Community
KU528942	B/Palermo/33/2013	Yamagata-like	2	06/02/2013	2 years	Female	Community
KU528944	B/Palermo/35/2013	Yamagata-like	2	06/02/2013	7 years	Male	Community
Pending	B/Genoa/4113/2013	Yamagata-like	2	07/02/2013	NA	NA	Hospital
KU528945	B/Palermo/36/2013	Yamagata-like	2	07/02/2013	6 years	Female	Community
KU528946	B/Palermo/37/2013	Yamagata-like	2	07/02/2013	4 years	Male	Community
KU528947	B/Palermo/38/2013	Yamagata-like	2	07/02/2013	5 years	Male	Community
KU528948	B/Palermo/40/2013	Yamagata-like	2	07/02/2013	7 years	Female	Community
KU528949	B/Palermo/41/2013	Yamagata-like	2	08/02/2013	6 years	Female	Community
KU528951	B/Palermo/43/2013	Yamagata-like	2	08/02/2013	55 years	Male	Hospital
Pending	B/Genoa/3613/2013	Yamagata-like	2	09/02/2013	NA	NA	Hospital
Pending	B/Genoa/3813/2013	Yamagata-like	2	09/02/2013	NA	NA	Hospital
KU528952	B/Palermo/44/2013	Yamagata-like	2	10/02/2013	31 years	Female	Hospital
KU528953	B/Palermo/45/2013	Yamagata-like	2	11/02/2013	11 years	Female	Community
KU528954	B/Palermo/46/2013	Yamagata-like	2	11/02/2013	9 years	Male	Community
KU528955	B/Palermo/47/2013	Yamagata-like	2	11/02/2013	12 years	Female	Community
KU528956	B/Palermo/48/2013	Yamagata-like	2	12/02/2013	5 years	Male	Community
KU528957	B/Palermo/49/2013	Yamagata-like	2	12/02/2013	9 years	Male	Community
KU528958	B/Palermo/50/2013	Yamagata-like	2	12/02/2013	9 years	Male	Community
KU528959	B/Palermo/51/2013	Yamagata-like	2	12/02/2013	4 years	Male	Community
KU528960	B/Palermo/52/2013	Yamagata-like	2	13/02/2013	63 years	Male	Community
KU528962	B/Palermo/54/2013	Yamagata-like	2	13/02/2013	6 months	Male	Community
KU528964	B/Palermo/56/2013	Yamagata-like	2	14/02/2013	12 years	Male	Community
KU528965	B/Palermo/57/2013	Yamagata-like	2	15/02/2013	6 years	Male	Hospital
KU528966	B/Palermo/58/2013	Yamagata-like	2	15/02/2013	8 years	Female	Community
KU528967	B/Palermo/59/2013	Yamagata-like	2	15/02/2013	10 years	Male	Hospital
Pending	B/Genoa/3913/2013	Yamagata-like	2	17/02/2013	NA	NA	Hospital
KU528968	B/Palermo/61/2013	Yamagata-like	2	18/02/2013	2 years	Male	Community
KU528969	B/Palermo/62/2013	Yamagata-like	2	18/02/2013	74 years	Female	Community
KU528970	B/Palermo/63/2013	Yamagata-like	2	18/02/2013	1 years	Female	Community
KU528971	B/Palermo/64/2013	Yamagata-like	2	18/02/2013	66 years	Female	Community
KU528972	B/Palermo/67/2013	Yamagata-like	2	18/02/2013	10 months	Female	Community
KU528973	B/Palermo/68/2013	Yamagata-like	2	18/02/2013	3 years	Male	Community
KU528974	B/Palermo/70/2013	Yamagata-like	2	19/02/2013	2 years	Female	Community
KU528975	B/Palermo/71/2013	Yamagata-like	2	19/02/2013	57 years	Female	Community
KU528976	B/Palermo/72/2013	Yamagata-like	2	19/02/2013	6 years	Male	Community
KU528977	B/Palermo/73/2013	Yamagata-like	2	19/02/2013	6 years	Male	Community
KU528978	B/Palermo/74/2013	Yamagata-like	2	19/02/2013	9 years	Male	Community
KU528980	B/Palermo/76/2013	Yamagata-like	2	20/02/2013	35 years	Female	Community
KU528981	B/Palermo/77/2013	Yamagata-like	2	20/02/2013	32 years	Male	Community
KU528982	B/Palermo/78/2013	Yamagata-like	2	21/02/2013	6 years	Male	Community
KU528983	B/Palermo/79/2013	Yamagata-like	2	21/02/2013	3 years	Female	Community
KU528984	B/Palermo/80/2013	Yamagata-like	2	21/02/2013	35 years	Female	Community
KU528985	B/Palermo/81/2013	Yamagata-like	2	22/02/2013	5 years	Male	Community
KU528986	B/Palermo/82/2013	Yamagata-like	2	22/02/2013	58 years	Male	Community
KU528987	B/Palermo/83/2013	Yamagata-like	2	22/02/2013	2 years	Female	Community
KU528988	B/Palermo/84/2013	Yamagata-like	2	22/02/2013	3 years	Female	Community
KU528990	B/Palermo/86-A/2013	Yamagata-like	2	23/02/2013	53 years	Female	Hospital
KU528993	B/Palermo/88/2013	Yamagata-like	2	25/02/2013	76 years	Female	Community

Table S1. *Cont.*

GENBANK ACCESSION NUMBER	VIRUS NAME	B LINEAGE	B CLADE	COLLECTION DATE	AGE	SEX	SOURCE
KU528994	B/Palermo/89/2013	Yamagata-like	2	25/02/2013	7 years	Male	Community
KU528995	B/Palermo/90/2013	Yamagata-like	2	25/02/2013	44 years	Female	Community
KU528996	B/Palermo/91/2013	Yamagata-like	2	25/02/2013	86 years	Male	Community
KU528997	B/Palermo/92/2013	Yamagata-like	2	25/02/2013	49 years	Female	Hospital
KU528998	B/Palermo/93/2013	Yamagata-like	2	25/02/2013	9 years	Male	Community
Pending	B/Genoa/4013/2013	Yamagata-like	2	25/02/2013	NA	NA	Hospital
KU528999	B/Palermo/95/2013	Yamagata-like	2	27/02/2013	13 years	Male	Community
KU529000	B/Palermo/96/2013	Yamagata-like	2	27/02/2013	77 years	Male	Hospital
KU529001	B/Palermo/97/2013	Yamagata-like	2	28/02/2013	5 years	Male	Community
KU529004	B/Palermo/100/2013	Yamagata-like	2	04/03/2013	5 years	Female	Community
KF700629	B/Palermo/101/2013	Yamagata-like	2	04/03/2013	18 years	Male	Community
KU529006	B/Palermo/102/2013	Yamagata-like	2	04/03/2013	10 years	Female	Community
KU529003	B/Palermo/99/2013	Yamagata-like	2	04/03/2013	6 years	Female	Community
KU529007	B/Palermo/103/2013	Yamagata-like	2	05/03/2013	44 years	Male	Hospital
KU529008	B/Palermo/104/2013	Yamagata-like	2	05/03/2013	54 years	Female	Hospital
KU529009	B/Palermo/105/2013	Yamagata-like	2	05/03/2013	1 years	Female	Community
KU529011	B/Palermo/107/2013	Yamagata-like	2	06/03/2013	6 years	Male	Community
KU529012	B/Palermo/108/2013	Yamagata-like	2	12/03/2013	6 years	Female	Community
KU529013	B/Palermo/109/2013	Yamagata-like	2	12/03/2013	4 years	Female	Community
KU529014	B/Palermo/110/2013	Yamagata-like	2	12/03/2013	29 years	Female	Community
KU529015	B/Palermo/111/2013	Yamagata-like	2	18/03/2013	3years	Female	Community
KU528991	B/Palermo/86-B/2013	Yamagata-like	2	20/03/2013	41years	Male	Community
KU529016	B/Palermo/112/2013	Yamagata-like	2	27/03/2013	45years	Female	Hospital
Pending	B/Genoa/0112/1213	Yamagata-like	3	28/12/2012	50years	Male	Hospital
KU528917	B/Palermo/2/2013	Yamagata-like	3	16/01/2013	4years	Female	Community
Pending	B/Genoa/2913/2013	Yamagata-like	3	24/01/2013	NA	NA	Hospital
Pending	B/Genoa/0613/1213	Yamagata-like	3	25/01/2013	47 years	Male	Hospital
Pending	B/Genoa/3013/2013	Yamagata-like	3	25/01/2013	NA	NA	Hospital
KU528963	B/Palermo/55/2013	Yamagata-like	3	13/02/2013	4 years	Male	Community
KU528989	B/Palermo/85/2013	Yamagata-like	3	22/02/2013	4 years	Male	Community
KU529010	B/Palermo/106/2013	Yamagata-like	3	06/03/2013	42 years	Female	Community
KU529019	B/Palermo/4/2015	Yamagata-like	3	26/01/2015	8 years	Female	Community
KU529020	B/Palermo/5/2015	Yamagata-like	3	27/01/2015	12 years	Female	Community
KU529021	B/Palermo/7/2015	Yamagata-like	3	05/02/2015	55 years	Male	Community
KU529022	B/Palermo/8/2015	Yamagata-like	3	10/02/2015	10 years	Male	Community
KU529023	B/Palermo/9/2015	Yamagata-like	3	13/02/2015	49 years	Male	Community
KU529024	B/Palermo/10/2015	Yamagata-like	3	13/02/2015	7 years	Male	Community
Pending	B/Genoa/09/2015	Yamagata-like	3	16/02/2015	66 years	Male	Hospital
KU529025	B/Palermo/11/2015	Yamagata-like	3	16/02/2015	37 years	Female	Community
KU529026	B/Palermo/12/2015	Yamagata-like	3	16/02/2015	39 years	Male	Community
KU529027	B/Palermo/13/2015	Yamagata-like	3	17/02/2015	8 years	Male	Community
Pending	B/Genoa/10/2015	Yamagata-like	3	20/02/2015	43 years	Male	Hospital
KU529028	B/Palermo/14/2015	Yamagata-like	3	23/02/2015	15 years	Male	Community
KU529029	B/Palermo/15/2015	Yamagata-like	3	24/02/2015	50 years	Male	Community
KU529030	B/Palermo/16/2015	Yamagata-like	3	25/02/2015	5 years	Female	Community
KU529031	B/Palermo/17/2015	Yamagata-like	3	26/02/2015	11 years	Female	Community
Pending	B/Genoa/11/2015	Yamagata-like	3	02/03/2015	41 years	Male	Hospital
KU529032	B/Palermo/18/2015	Yamagata-like	3	02/03/2015	11 years	Male	Community
Pending	B/Genoa/12/2015	Yamagata-like	3	03/03/2015	57 years	Female	Hospital
KU529033	B/Palermo/19/2015	Yamagata-like	3	03/03/2015	15 years	Female	Hospital
Pending	B/Genoa/14/2015	Yamagata-like	3	04/03/2015	90 years	Female	Hospital
KU529036	B/Palermo/23/2015	Yamagata-like	3	10/03/2015	4 years	Male	Community
Pending	B/Genoa/15/2015	Yamagata-like	3	11/03/2015	69 years	Male	Hospital
KU529038	B/Palermo/25/2015	Yamagata-like	3	12/03/2015	17 years	Male	Community
KU529039	B/Palermo/26/2015	Yamagata-like	3	12/03/2015	40 years	Male	Community
KU529040	B/Palermo/27/2015	Yamagata-like	3	12/03/2015	7 years	Male	Community
KU529041	B/Palermo/28/2015	Yamagata-like	3	12/03/2015	40 years	Female	Community
Pending	B/Genoa/16/2015	Yamagata-like	3	16/03/2015	90 years	Female	Hospital
KU529042	B/Palermo/29/2015	Yamagata-like	3	16/03/2015	46 years	Male	Community
KU529043	B/Palermo/30/2015	Yamagata-like	3	16/03/2015	2 years	Male	Hospital
KU529045	B/Palermo/32/2015	Yamagata-like	3	18/03/2015	32 years	Male	Community
KU529046	B/Palermo/33/2015	Yamagata-like	3	20/03/2015	37 years	Female	Community
KU529051	B/Palermo/38/2015	Yamagata-like	3	30/03/2015	8 years	Male	Community
KU529052	B/Palermo/39/2015	Yamagata-like	3	30/03/2015	67 years	Male	Community
KU529055	B/Palermo/42/2015	Yamagata-like	3	30/03/2015	2 years	Female	Community
KU529056	B/Palermo/43/2015	Yamagata-like	3	30/03/2015	6 years	Male	Community
KU529057	B/Palermo/45/2015	Yamagata-like	3	01/04/2015	7 years	Male	Community
KU529058	B/Palermo/47/2015	Yamagata-like	3	01/04/2015	11 years	Male	Community

Table S1. *Cont.*

GENBANK ACCESSION NUMBER	VIRUS NAME	B LINEAGE	B CLADE	COLLECTION DATE	AGE	SEX	SOURCE
KU529059	B/Palermo/48/2015	Yamagata-like	3	03/04/2015	9 years	Male	Community
KU529060	B/Palermo/49/2015	Yamagata-like	3	03/04/2015	55 years	Male	Community
KU529061	B/Palermo/50/2015	Yamagata-like	3	04/04/2015	31 years	Female	Community
KU529062	B/Palermo/51/2015	Yamagata-like	3	05/04/2015	37 years	Male	Hospital
Pending	B/Genoa/17/2015	Yamagata-like	3	29/04/2015	87 years	Female	Hospital
KU529064	B/Palermo/53/2015	Yamagata-like	3	19/05/2015	80 years	Male	Hospital
Pending	B/Genoa/18/2015	Yamagata-like	3	22/05/2015	90 years	Female	Hospital
KU529017	B/Palermo/1/2015	Yamagata-like	3A	19/01/2015	68 years	Female	Community
KU529018	B/Palermo/2/2015	Yamagata-like	3A	21/01/2015	30 years	Female	Community
KU529034	B/Palermo/21/2015	Yamagata-like	3A	10/03/2015	12 years	Female	Community
KU529049	B/Palermo/36/2015	Yamagata-like	3A	25/03/2015	6 years	Male	Community
KU528913	B/Palermo/2/2011	Yamagata-like	Non determined	14/01/2011	51 years	Male	Hospital
Pending	B/Genoa/01/2014	Yamagata-like	Non determined	25/02/2014	48 years	Male	Hospital

NA: not available.

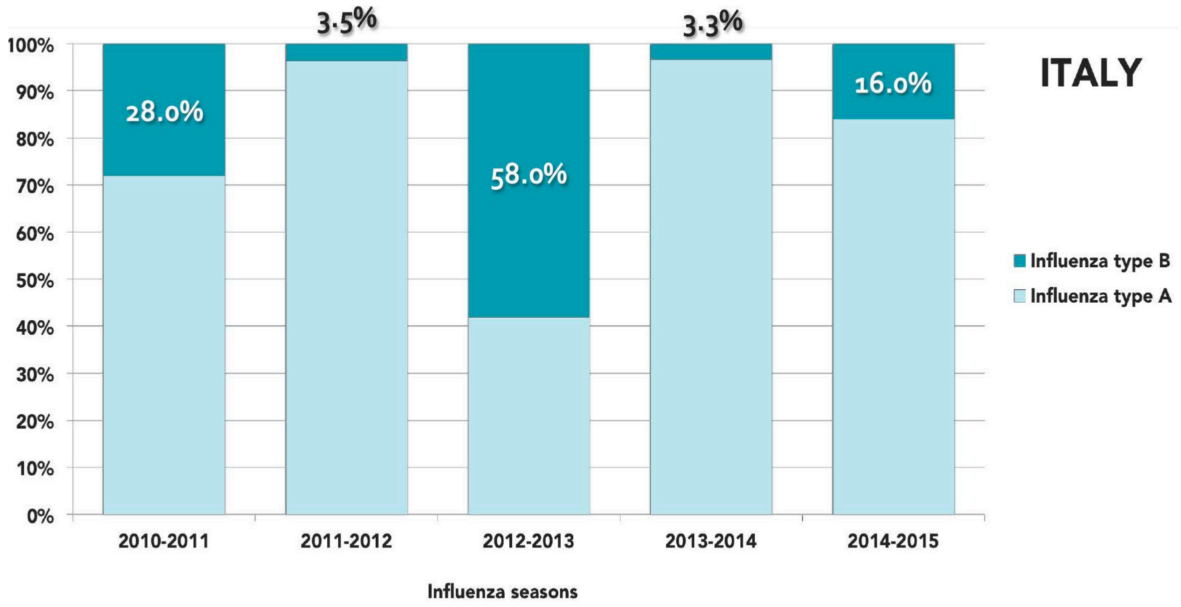

Figure S1. Relative proportions of influenza virus types detected in Italy, according to seasonal epidemics. Percentages reported in the figure refer to influenza B. Source: virological data from Italian Surveillance Network (INFLUNET).

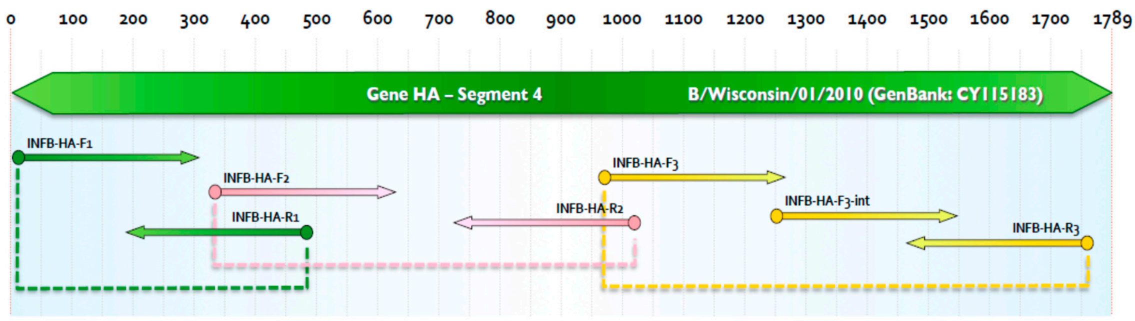

Fragment	Primer	Sequence
1b	INFB-HA-F1	5′-ATG AAG GCA ATA ATT GTA CTA CTC-3′
1b	INFB-HA-R1	5′-GGC AAG AYC CTG AGG TTC C-3′
2b	INFB-HA-F2	5′-CTT TCC TAT AAT GCA CGA CAG AAC-3′
2b	INFB-HA-R2	5′-CAT ATT GGG CAA TTT CCT ATG GC-3′
3b	INFB-HA-F3	5′-AGC AAG CCT TAC TAC ACA GG-3′
3b	INFB-HA-F3-int	5′-ATG AAC TCC ACA ACG AAA TAC TCG-3′
3b	INFB-HA-R3	5′-CCT TAT AGA CAG ATG GAG CAA G-3′

Figure S2. Schematic representation of Influenza B gene HA sequencing.
